# Care of ophthalmic surgical instruments

**Published:** 2011-12

**Authors:** Sue Stevens, Ingrid Mason

**Affiliations:** Former Nurse Advisor, Community Eye Health Journal, International Centre for Eye Health, London School of Hygiene and Tropical Medicine, Keppel Street, London WC1E 7HT, UK.; CBM Medical Advisor, PO Box 58004, 00200 City Square, Ring Road Parklands, Nairobi, Kenya.

**Figure:**
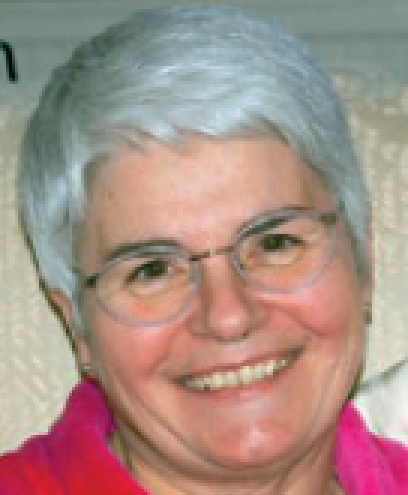
Sue Stevens

**Figure:**
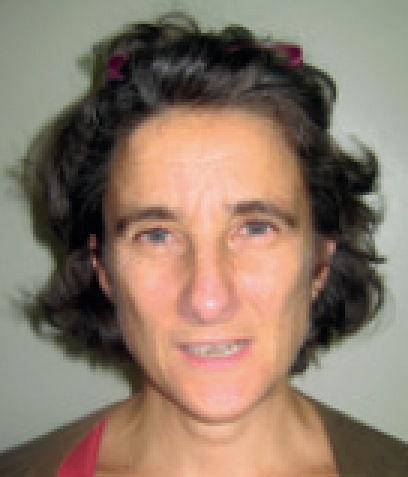
Ingrid Mason

**Handling and safety**

**Figure:**
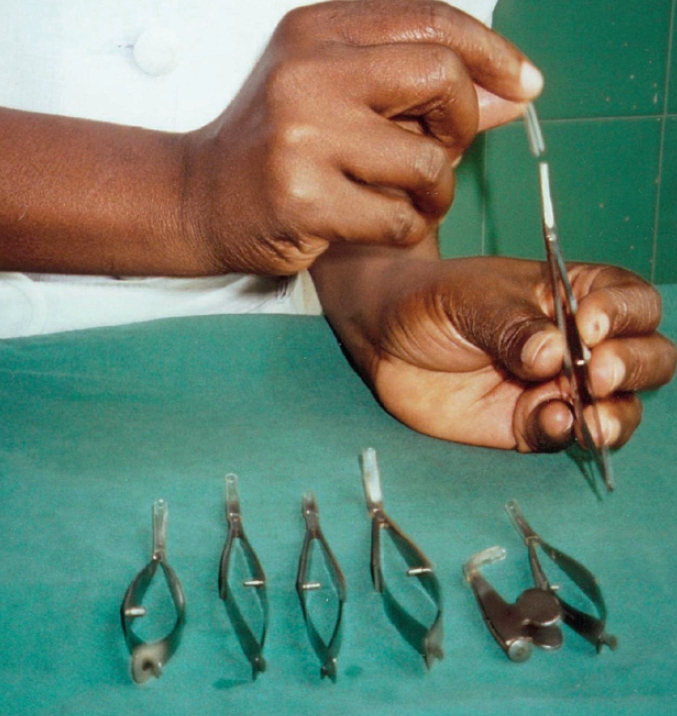

**Sharps**

Protect the tips of all sharp instruments with silicone or rubber tubing.

Intravenous infusion tubing or tubing from ‘butterfly’ intravenous needles may be used.

**Remember!**

- Never re-sheath a disposable needle
- Always use artery forceps to remove a blade from a Bard Parker handle
- Provide a gallipot on the theatre trolley to collect used needles and blades
- Do not touch the tips of any instrument
- Never throw an instrument down

**Needles**

Discard used needles immediately after use.

Place in a receptacle used only for this purpose.

Do not over-fill.

Preferably use small receptacles and dispose of them daily.

Seal and incinerate the receptacle when almost full.

**Figure:**
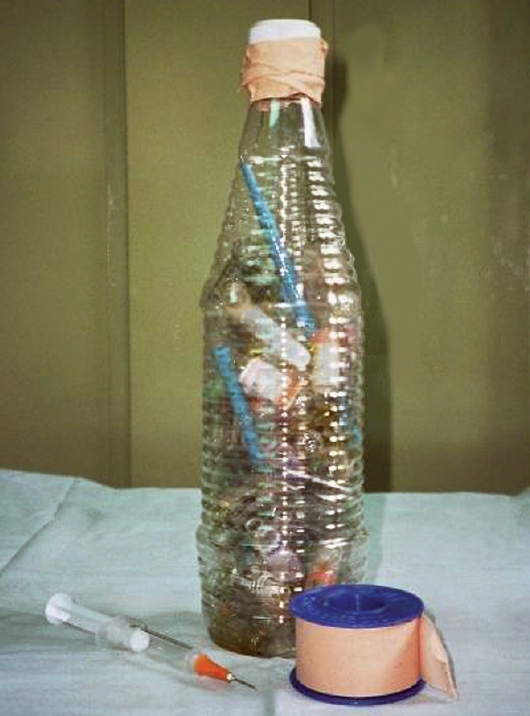

**Maintenance**

**Remember! All of these maintenance tasks must be done before you sterilise the instruments.**

**Cleaning**

Ideally, instruments must be cleaned immediately after surgery (within 20 minutes). If this is not possible, place them in a pH neutral enzymatic solution or at the very least cover them with a moistened towel to prevent blood, tissue, and saline from drying and caking on the instruments.

Use a soft toothbrush and warm soapy water to thoroughly clean each instrument individually and in its **open** position.

Water should be warm, not hot. Hot water causes blood to clot (coagulate) faster, making it harder to remove.

Distilled water is preferable since regular water can leave mineral deposits.

**Figure:**
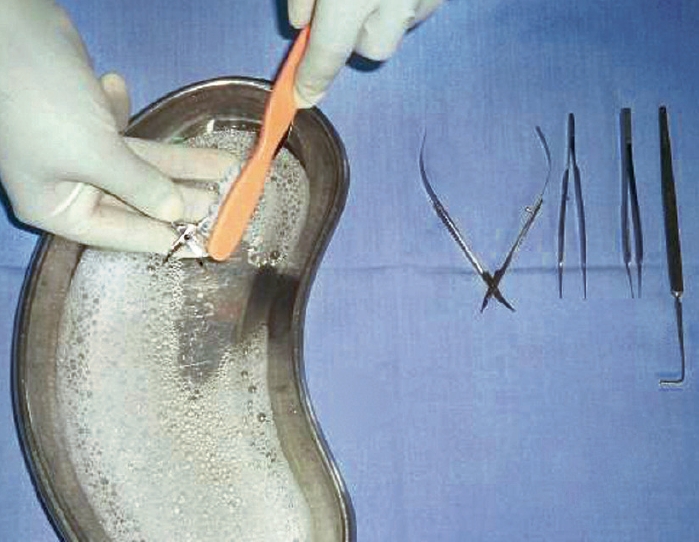

**Lubricating hinged instruments after cleaning**

Use a lubricant immediately after cleaning hinged instruments to prevent rust and stiff joints.

Ideally, use water-based synthetic lubricants as these are designed to be compatible with sterilisation. Oil-based lubricants (mineral or silicone) can coat micro-organisms and prevent penetration of steam, preventing adequate sterilisation.

If water-based lubricants are not available, ordinary sewing machine oil is acceptable.

**Figure:**
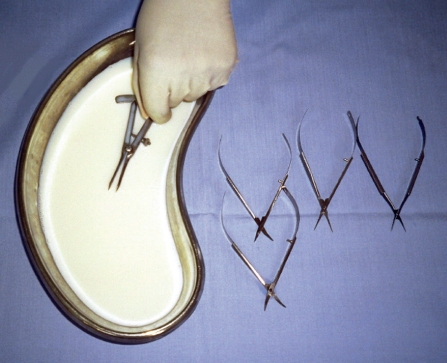

If you are using water-based lubricants, dip instruments and allow the lubricant to drain off (pictured). **Do not leave to soak, and never put cannulae in lubricant.**

If you are using sewing-machine oil, use a 2 ml syringe and a 21-gauge needle to draw up the oil and a 25-gauge needle to apply oil to the joints. Use a piece of gauze to carefully wipe away any surplus oil.

If any hinged instruments are stored, you must lubricate them at least once a week.

**Drying**

**Figure:**
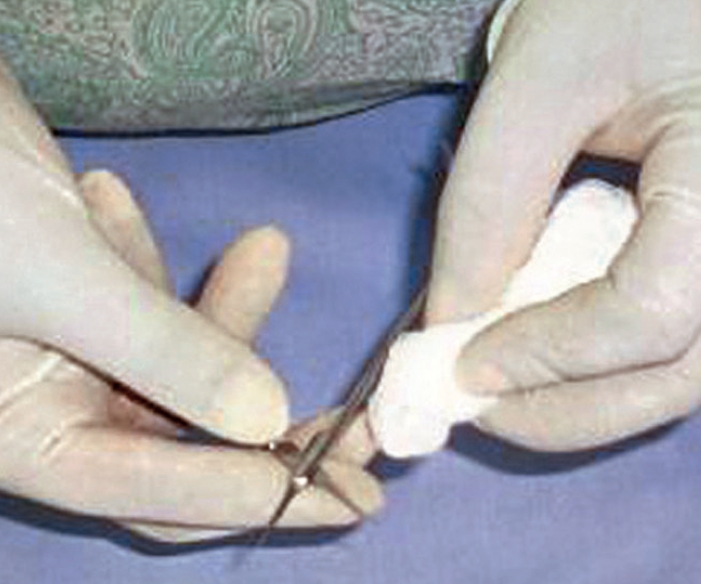

Thoroughly dry instruments before storing or sterilising them. Dry gauze (used cautiously) or a hairdryer may be used.

**Inspecting instruments**

**Figure:**
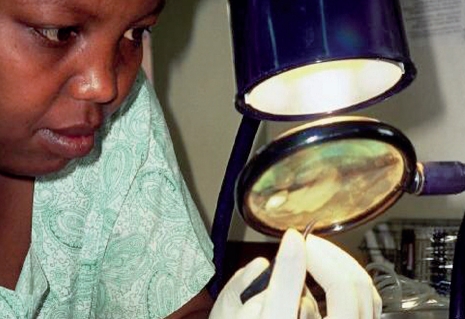

Inspect instruments for alignment and sharpness under a good light and magnification.

**Figure:**
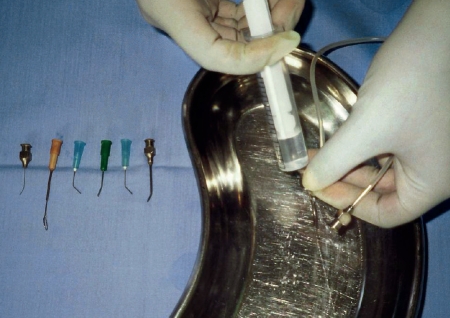

Inspect cannulae to ensure they are not obstructed by flushing through with clean, warm water.

**Storage, transport, and security**

**Remember!** Silicone or rubber protectors must be used on sharp instruments when in storage or transit.

**Shelves**

**Figure:**
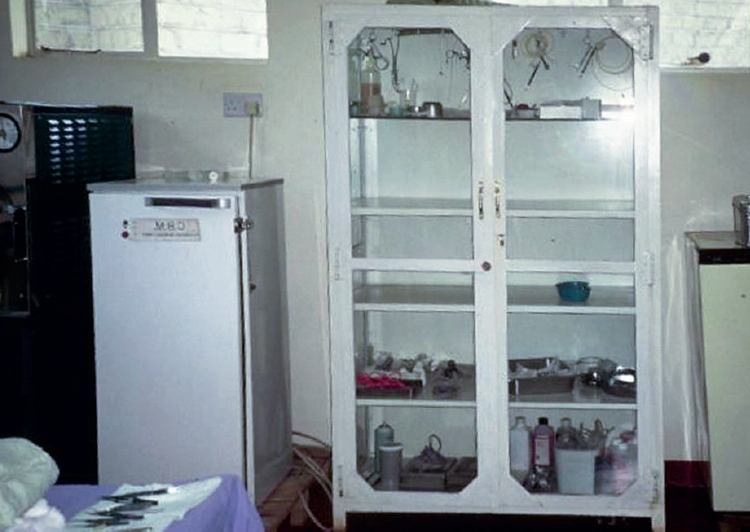

Glass shelves in a lockable cupboard provide for secure storage and easy checking.

**Never** pile instruments on top of each other.

A well-ventilated room is recommended.

**Trays**

**Figure:**
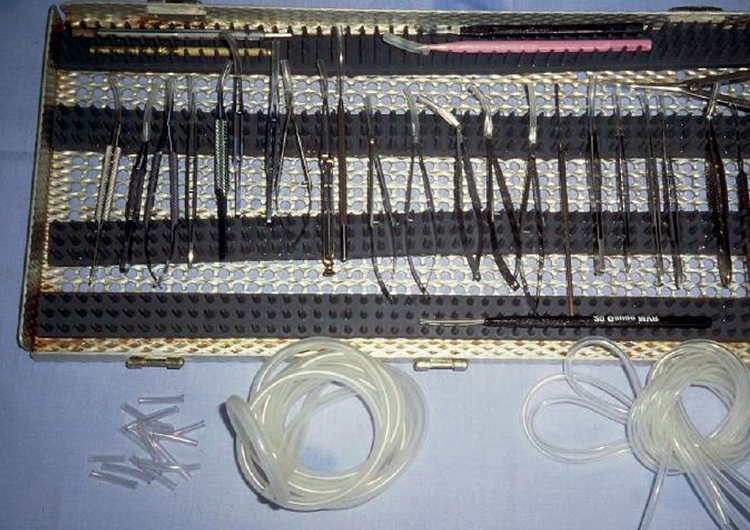

Each individual slot in the tray holds a single instrument.

Instruments must not touch each other.

The tray can be used for storage, transportation, and during some sterilisation procedures.

**Cases**

**Figure:**
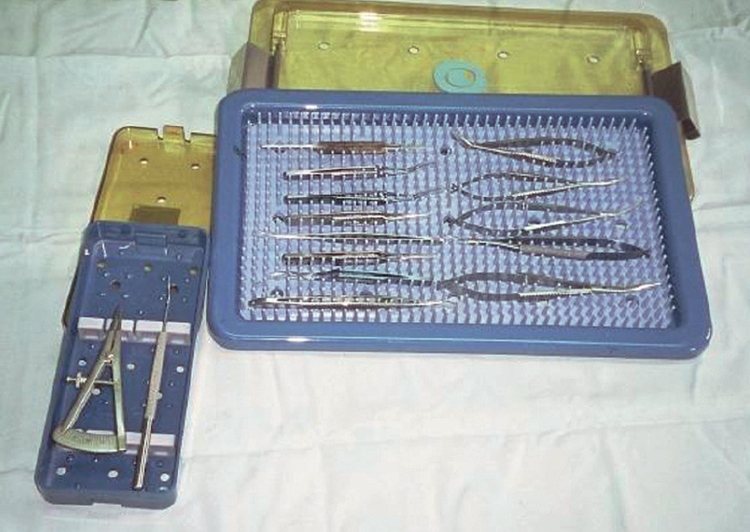

Cases may be of metal or plastic and contain a protective silicone mat.

Cases can be used for storage, transportation, and during some sterilisation procedures.

**Rolls**

**Figure:**
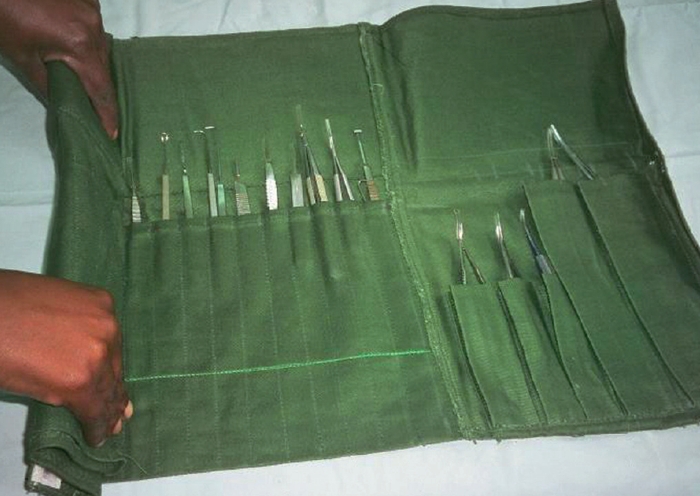

Rolls, made of strong fabric, are inexpensive. Each pocket holds a single instrument.

Secure the roll with ribbon or cord, not elastic, as elastic can degrade in heat.

Use rolls only for storage and transportation of instruments, not for any other purpose.

